# Medium versus difficult visual search: How a quantitative change in the functional visual field leads to a qualitative difference in performance

**DOI:** 10.3758/s13414-019-01787-4

**Published:** 2019-07-02

**Authors:** Johan Hulleman, Kristofer Lund, Paul A. Skarratt

**Affiliations:** 1 School of Biological Sciences, The University of Manchester, Oxford Road, Manchester, M13 9PL UK; 2 Department of Psychology, University of Hull, Hull, UK

**Keywords:** Visual search, Eye movements, Task difficulty, Functional Visual Field (FVF), Guided Search, Attentional Engagement Theory (AET), Feature Integration Theory (FIT)

## Abstract

The dominant theories of visual search assume that search is a process involving comparisons of individual items against a target description that is based on the properties of the target in isolation. Here, we present four experiments that demonstrate that this holds true only in difficult search. In medium search it seems that the relation between the target and neighbouring items is also part of the target description. We used two sets of oriented lines to construct the search items. The cardinal set contained horizontal and vertical lines, the diagonal set contained left diagonal and right diagonal lines. In all experiments, participants knew the identity of the target and the line set used to construct it. In difficult search this knowledge allowed performance to improve in displays where only half of the search items came from the same line set as the target (50% eligibility), relative to displays where all items did (100% eligibility). However, in medium search, performance was actually poorer for 50% eligibility, especially on target-absent trials. This opposite effect of ineligible items in medium search and difficult search is hard to reconcile with theories based on individual items. It is more in line with theories that conceive search as a sequence of fixations where the number of items processed during a fixation depends on the difficulty of the search task: When search is medium, multiple items are processed per fixation. But when search is difficult, only a single item is processed.

**Electronic supplementary material:**

The online version of this article (10.3758/s13414-019-01787-4) contains supplementary material, which is available to authorized users.

## Introduction

Visual search is everywhere, whether you are trying to find a friend amongst disembarking passengers or looking for a street name sign to establish your whereabouts. Search tasks are widely studied in their own right, but also used in a variety of fields ranging from memory and cognitive control to clinical assessment. In most searches, failure to find the target is without serious consequences. Yet when airport screeners search for threats or radiologists search for lesions, success matters. To improve performance in these important visual searches, and to understand visual search performance in general, it is critical to establish underlying processes and mechanisms.

Feature Integration Theory (FIT), Guided Search and Attentional Engagement Theory (AET) are by far the most successful theories of visual search. Although they were developed several decades ago, they are still cited some 100–300 times a year. This triumvirate also dominates textbook descriptions of visual search (Hulleman & Olivers, 2017b).

In this paper, we test the fundamental assumption of these three models that search is always based on the properties of the target in isolation, irrespective of the difficulty of the search task. But before we describe our experiments, we would like to give a detailed description of the inner workings of the models, to clearly establish how they operate and what they do and do not predict. In particular in relation to our chosen manipulation: The introduction of items that clearly cannot be the target in search tasks that are considered to be completely item-by-item by all three.

FIT (Treisman & Gelade, 1980) is the oldest theory. Arguably, it kick-started the continuing interest in visual search. According to Treisman and Gelade (1980), there is a fundamental distinction between serial search (where RTs increase with the number of items in the display) and parallel search (where RTs are independent). Inspired by the work of Zeki (1976) on the visual cortex in rhesus monkeys, Treisman and Gelade (1980) proposed that the information from search displays is split into several feature maps (colour, orientation, motion).

When search is parallel, the presence or absence of a target can be established by inspection of the relevant feature map. For instance, in search for a red target amongst green distractors there will only be activity in the red colour map when the target is present. When the target is absent, there is no activity. This holds true irrespective of the number green distractors in the display. Consequently, when RTs are plotted as a function of the number of items, the slope will be flat (~0 ms/item), both for present trials and absent trials. In serial search however, inspection of the feature maps is not sufficient to establish target presence.

When searching for red-horizontal amongst green-horizontal and red-vertical, there will always be activity in all four relevant feature maps (red, green, horizontal and vertical), both when the target is present and when it is absent. Treisman and Gelade (1980) proposed that this necessitates a second, serial step. In a master map of locations, attention is applied to one item at a time to bind the features from the different maps and compare them with a description of the target. This binding process continues until the item with the correct feature combination is found or until all items have been rejected as distractors. The serial application of attention to individual items explains why RTs increase with the number of items in this type of search. When there is a target, on average it will be found after half of the items have been inspected. So, the more items there are, the longer this will take. It also explains why average RTs for absent trials increase more steeply: here, all items have to be inspected before a response that the target is absent is possible. In a plot of RTs against number of items, serial search has a clear slope for the present trials (~20 ms/item) and the slope for absent trials is about twice as steep (~40 ms/item).

In the wake of Treisman and Gelade (1980), the qualitative distinction between serial and parallel search became a key bone of theoretical contention. After a set of results that seemed to question the predictions made by FIT (e.g. Egeth, Virzi & Garbart, 1984; McLeod, Driver & Crisp, 1988; Nakayama & Silverman, 1986), both AET and Guided Search called into question the qualitative nature of the distinction and proposed that the difference between search with serial and parallel slopes is merely quantitative.

Guided Search (Wolfe, Cave & Franzel, 1989; Wolfe, 1994) is most closely related to FIT. The crucial difference is that Guided Search no longer assumes that it is possible to inspect individual feature maps. Rather, feature information is captured by broadly tuned channels (steep, shallow, left and right for orientation; red, yellow, green and blue for colour). Subsequently, the feature information is pooled into an activation map. Attention is applied serially to this activation map. The item with the highest activation is inspected first. If the item is the target, search can be terminated with a target-present response. If it is not, the current item is inhibited and the item with the next highest activation will be selected and inspected. This cycle of selection, inspection and inhibition continues either until the target has been found or until there are no items left with activation above the activation threshold. In the latter case, search will be terminated with a target-absent response.

What distinguishes serial from parallel search in Guided Search is that in parallel search the target always has the highest activation. This means that it will be the first to be selected, irrespective of the number of distractors. Guided Search achieves this by a combination of bottom-up and top-down activation. Bottom-up activation reflects how much an item stands out from its neighbours and allows Guided Search to model odd-one-out searches. Guided Search uses top-down activation to model conjunction searches where the RTs suggest that the target has an enhanced chance of selection.

For instance, search for green horizontal amongst red horizontal and green vertical yields search slopes (~10 ms/item on target present trials) that fall between complete independence of the number of items and exhaustive serial search. According to Guided Search, this is due to top-down activation. Because observers know they are looking for green horizontal, they selectively tune in to the green colour channel and the horizontal orientation channel. Consequently, the target will have twice the activation level of distractors, since it receives activation from both channels, whereas distractors only receive activation from one channel (either green or horizontal). This allows the target to be found faster, since it will always be amongst the items with the highest activations. (It is not necessarily the item with the highest activation, since there is also noise in the activation map.)

When neither bottom-up activation nor top-down activation can be used to boost the activation of the target, search becomes unguided. The prototypical case for this is T versus L search (Wolfe et al., 1989; Wolfe, 1994, 2007). Since all items contain the same lines, none of them stands out and there is no bottom-up activation. Nor is there a possibility of top-down activation, as tuning in to a particular orientation channel does not selectively enhance the activation of the target relative to the distractors.

AET (Duncan & Humphreys, 1989) was created in the same year as Guided Search. It also dispenses with the qualitative distinction between serial and parallel search, but otherwise has a rather different characterisation of the visual search task. Although it is probably best known for introducing the concepts of target-nontarget similarity and nontarget-nontarget similarity (T-N similarity and N-N similarity, respectively), the twin fundament of AET is formed by the *search template* and *weight linkage*. According to AET, items need access to Visual Short-Term Memory (VSTM) to become the focus of behaviour.

Access to VSTM is strictly limited and whether an item gains access or not depends on its selection weight. The higher this selection weight, the higher the probability that the item will gain access to VSTM. The selection weight is determined by an item’s match against the search template (an advance specification of the information sought). If there is a good match the item’s selection weight increases; if there is not, its selection weight decreases. This is where weight linkage becomes important. The more similar items are, the more a change to one item’s selection weight will spread to those similar items. Thus, the search template and weight linkage underlie the influence of T-N similarity and N-N similarity. The less nontargets look like the target, the more the target will be the only item with a good match to the search template and the more likely it is to gain access to VSTM, speeding up search. The more nontargets look like each other, the easier it is to reject them together, because the change in selection weight spreads amongst them. Again, this will speed up search.

After their initial specification, all three theories have undergone changes. Some relatively minor, some quite substantial. The results that prompted the formulation of AET and Guided Search (Egeth et al., 1984; McLeod et al., 1988; Nakayama & Silverman, 1986), also elicited several modifications to FIT. Treisman and Souther (1985) discussed the case where target and distractors share the feature that distinguishes between them, but where the target has more of it (e.g. closedness). They proposed that a response pooled across several items might be able to distinguish between a group of items that contains a target and a group of items that does not. Treisman and Sato (1990) further extended FIT by adding feature inhibition. This inhibition is generated in feature maps that code for nontarget features and removes activity from the master map at locations where there are distractors. This results in faster search for conjunctions, since the reduction in activity allows pooling over larger groups of items.

Nevertheless, it should be pointed out that there are clear limits to the pooling approach in FIT, as illustrated by the case of T versus L search: *“[…] search is serial for a conjunction of the same two features in different spatial arrangements (e.g., Ts among Ls in four randomly varying orientations). If we assume that Ts and Ls are both composed of one horizontal and one vertical line, then neither has any unique feature through which inhibition could be controlled, so that item-by-item search is required”* (Treisman & Sato, 1990, p.476).

Three major changes were made to Guided Search when it was updated to version 4.0 (Wolfe, 2007). The first was a new way to compute the activation map. In Guided Search 4.0 this is now a weighted sum of bottom-up activation, top-down activation and noise. The bottom-up activation for a particular item is derived from pairwise comparisons with all of the other items in the display. For each of the categorical channels within a feature dimension (steep, shallow, left and right for orientation; red, yellow and green for colour) the difference in response is determined. The maximum of these differences is chosen and scaled by the distance between the items. The bottom-up activation for a particular item is then the sum of all the scaled maximum differences. Top-down activation for an item is a weighted sum of its own response in the various categorical channels. Top-down guidance is implemented by giving a particular channel within a dimension (one each for colour and orientation) a weight of 1.0 and the other channels in this dimension a weight of 0. As before, this allows Guided Search 4.0 to predict the correct search slopes for green horizontal amongst green vertical and red horizontal, since the green horizontal target will be the only item in the activation map receiving top-down activation from both the horizontal and the green channels.

As stated above, the activation map now reflects the weighted sum of bottom-up activation, top-down activation and noise. Although Guided Search 4.0 does not allow for bottom-up activation to be completely ignored (i.e. have a weight of 0), it does assume that top-down guidance can be the determining factor for search performance. Even in displays where there are multiple orientations or colours.

The second change in Guided Search 4.0 was a reinterpretation of search slopes and the introduction of a parallel component into its architecture. Originally, Guided Search interpreted search slopes as an estimate of the time it takes to determine whether or not an item is a target. However, typical search slopes are in the range of 20-40 ms/item. This is much shorter than the lowest estimates of processing time derived from other methods (Duncan, Ward & Shapiro, 1994; Theeuwes, Godijn & Pratt, 2004). In Guided Search 4.0 (Wolfe, 2007), the search slope is now seen as the rate at which items enter a parallel diffusor that determines whether they are the target or a distractor. The diffusor takes between 150 and 300 ms to reach a decision boundary and several items may be processed in parallel. Wolfe (2007) used the carwash as a metaphor. Cars enter the carwash one-by-one, but several cars can be washed simultaneously. In the same way, attention selects items one-by-one and delivers them to the parallel processing stage that works on multiple items.

As a consequence, Guided Search 4.0 is now also able to account for the typical pattern of variability in visual search RTs. It correctly predicts that RTs in target-absent trials are more variable than those in target-present trials for 2 versus 5 search (Guided Search 2.0 predicted the opposite).

The third change was that Guided Search no longer assumes perfect memory for rejected distractors. Rather, every time a new item is selected, there is a 75% chance that the inhibition of a previously rejected distractor is lifted. On average this means that there are three inhibited items at any one time during a search.

In response to experiments by Treisman (1991), Duncan and Humphreys (1992) went into more detail about the specifics of template matching and spreading of suppression in AET. They argued that special weight is given to identity on a particular feature. Moreover, the influence of identity is larger on matches with the search template than on matches between nontargets. In a further development, Humphreys and Müller (1993) and Müller, Humphreys and Donelly (1994) presented SERR (SEarch via Recursive Rejection) as a computational implementation of several aspects of AET.

In SERR, there are template units that code the target and distractors used in the simulation. When the target template fires, a target-present decision is made. If a distractor template fires, it will lead to suppression of all the locations with distractors of this kind. Typically, the distractor templates will fire first, since it is easier for them to reach the firing threshold: there are multiple distractors, but only one target. So, for SERR, finding the target involves one or more rounds of rejection of distractors to leave the target standing; hence its name. It should be noted that SERR has one idiosyncrasy: a single pass results in very high error rates, especially at larger display sizes. To combat this, simulations are rerun on miss trials until the error rates have become comparable to those found for human observers.

In spite of all their differences, AET, FIT and Guided Search have several important characteristics in common. First, they ultimately conceive visual search as a process in which individual items are compared to a target description until the target is found or there are no viable candidates left. Second, they do not take the physiological constraints of the retina into account. Neither do they explicitly allow for eye movements. Rather, they all rely on a fast parallel process that yields the input description used in the search process. Third, their theoretical focus is on understanding the difference between searches with flat slopes and searches with steeper slopes. This means that they all propose a level of difficulty beyond which search becomes a serial process of inspecting one item after another until the target has been found or all have been rejected as distractors. For FIT and Guided Search, this stage is reached when target-present slopes are 20–30 ms/item and target absent slopes are 40–60 ms/item (with T versus L search as a typical example). For AET the values are slightly higher: 38–45 ms/item for target-present and 56–71 ms/item for target-absent (with L vs. Ls rotated 90° clockwise/counter-clockwise as a typical example).

These commonalities between AET, FIT and Guided Search prompted Hulleman and Olivers (2017a) to categorize them as item-based approaches. Hulleman and Olivers (2017a) contrasted them with what they termed fixation-based approaches. Fixation-based approaches allocate the determining role for visual search performance to eye movements and the properties of the retina (e.g. the Target Acquisition Model (TAM); Zelinsky, 2008, 2012; Zelinsky, Adeli, Peng & Samaras, 2013, and the Area Activation Model (AAM); Pomplun, Rheingold & Shen, 2003; Pomplun, 2007).

Interest in the influence of retinal physiology and eye movements on visual search can be traced back to Engel (1977), who investigated the relationship between target discrimination accuracy in the periphery and the number of eye movements made in visual search. There are several other milestone studies. Geisler and Chou (1995) used peripheral discrimination ability to create a model that predicted the RTs in visual search. Zelinsky and Sheinberg (1995) demonstrated that there was a relationship between the number of eye movements and manual RTs in visual search. Findlay and Gilchrist (2003) proposed that retinal limitations rather than central attentive processes are the major factor that determines visual search performance. Building on this fixation-based tradition, Hulleman and Olivers (2017a) proposed a framework for visual search that combined five elements with a substantial pedigree in the literature:

1. *Functional Visual Field (FVF).* This is the area of the visual field, centred on fixation, in which it is possible to reach a decision about the presence or absence of a target. It is the result of the interaction between task demands and retinal and cortical limitations. If a distinction can be made far into the periphery, the FVF will be large and cover multiple items (perhaps even the entire search display). When a distinction is very difficult, the FVF will be small and may cover only a single item. Similar proposals were made earlier: for example visual span (O’Regan, Lévy-Schoen, & Jacobs, 1983; Jacobs, 1986) and useful field of view (UFOV, Ball, Beard, Roenker, Miller & Griggs, 1988).
2. *Parallel processing of items within FVF*. Hulleman and Olivers (2017a) envisaged that all items within the FVF are processed simultaneously. They proposed that pooled statistics (Rosenholtz, Huang, Raj, Ballas & Ilie, 2012) are computed across the FVF. It is on the basis of these statistics, rather than on the basis of the properties of a single item, that the decision about target presence is reached (see also R3 in Hulleman & Olivers, 2017b). Something comparable can be found in AAM (Pomplun et al., 2003; Pomplun, 2007)
3. *Fixed fixation duration*. The framework assumes that the duration of a fixation is constant at around 250 ms, irrespective of the difficulty of the search task. This assumption was based on the work of Findlay (1997), Gilchrist and Harvey (2000), Hooge and Erkelens (1996) and Over, Hooge, Vlaskamp and Erkelens (2007).
4. *Limited avoidance of previously fixated areas.* During visual search, the last four areas of the search display that have been fixated are remembered. They are avoided when a new part of the search display is selected. When reaching a decision takes longer, these areas again become candidates for a new fixation. Since the FVF may cover multiple items, many more than four items may be unavailable for selection at a particular moment during visual search. This assumption of limited memory follows Gilchrist and Harvey (2004) and McCarly, Wang, Kramer, Irwin and Peterson (2003).
5. *A stopping rule*. As extensively discussed in Chun and Wolfe (1996), most participants do not process all items in the search display before they respond. Based on an analysis of the data of Young and Hulleman (2013), the framework assumes that search will be terminated with a target-absent response once at least 85% of the search display has been covered.

According to the framework of Hulleman and Olivers (2017a), the overall RT for a visual search display is the result of the number of fixations needed to reach a decision. This number is in turn determined by the size of the FVF. When the FVF is large, only a few fixations will be needed to reach a decision and RTs will be fast. When the FVF is small, many fixations will be needed and RTs will be slow.

Hulleman and Olivers (2017a) ran simulations of their framework for three types of visual search and allowed only the size of the FVF to vary. They found that an FVF that covered up to 30 items worked best for easy search (/ vs. |); that medium search (T vs. L) needed an FVF size that covered up to seven items and that difficult search (a configuration of a larger and a smaller square amongst rotations of this configuration) was best modelled with an FVF that covered only a single item. The simulations captured important qualitative aspects of all three search difficulties in terms of RTs, error rates, and distribution of RTs.

Of particular note is that the simulation successfully captured some qualitative differences between difficult search on the one hand and the two easier searches on the other. Whereas RTs in target-present trials are *less* variable than those in target-present trials in easy and medium search, this pattern reverses in difficult search. Here, RTs in target-present trials are actually *more* variable than those in target-absent trials. This suggests that the quantitative reduction in FVF size to only a single item in difficult search results in a qualitative change in search behaviour relative to easier searches. The size reduction of the FVF in difficult search means that it goes from a process that is a combination of parallel (within a fixation) and serial (successive fixations) to a process that is purely serial.

This qualitative difference between easier and difficult search sets up an interesting clash of predictions between the item-based theories and the FVF-framework. Because all three item-based theories are focused on explaining searches with flat slopes, none of them makes a principled distinction between different kinds of serial search. They therefore predict qualitatively similar behaviour for medium and difficult search. This holds especially true for FIT and Guided Search who interpret a T versus L search as *“item-by-item”* (Treisman & Sato, 1990, p. 476) and as having *“no guidance based on basic feature information”* (Wolfe, 1994, p.225).This *“lack of guidance makes the search inefficient”* (Wolfe, 2007, p.206). The same characterisation applies *a fortiori* to difficult search. The suggestion that the feature information needed for faster search is lacking, also points to a way of testing the prediction of similar behaviour in medium and difficult search; that is, to introduce a feature that does provide this information.

Perhaps surprisingly, given the almost 40 years that have passed since FIT was published in 1980, the number of undoubted features is quite small. Horowitz and Wolfe (2017) list exactly four: colour, orientation, motion and size (including length, spatial frequency, and apparent size). In the present experiments we used orientation, because we wanted to ensure that the feature is used during the visual search process itself, rather than during the initial, parallel operation that delivers the representation on which it operates.

For colour, this was something already discussed as a possibility by Egeth et al. (1984, p.39): “*[…] it would appear that with a large asymmetry in the number of two distractor types that figure-ground segregation may be occurring, with all of the elements forming the ground being rejected in parallel*”. For motion a similar type of operation has been proposed. McLeod et al. (1988) reported flat search slopes when participants had to find a moving X amongst moving O’s and static X’s. According to McLeod et al. (1988) this was the consequence of the activity of a movement filter that only allows moving items to pass. This movement filter was envisioned to act before the application of attention in the visual search display as becomes clear from McLeod, Driver, Dienes and Crisp (1991). McLeod et al. (1991) suggested that attention works within the filter to pick out a particular direction of motion *after* the filter itself has let through all moving items.

Since size may be vulnerable to spatial frequency filtering, orientation remains as the most suitable choice. Its status as a feature is undoubted and it has been part of AET, FIT and Guided Search since their inception. Indeed, orientation is part of the list of separable dimensions in Treisman and Gelade (1980), is used in Wolfe et al. (1989), and figures prominently in Duncan and Humphreys (1989). Orientation is also one of the two features implemented in Guided Search (Wolfe, 1994, 2007) and SERR has orientation-sensitive filters (Müller & Humphreys, 1993).

**Fig. 1:**
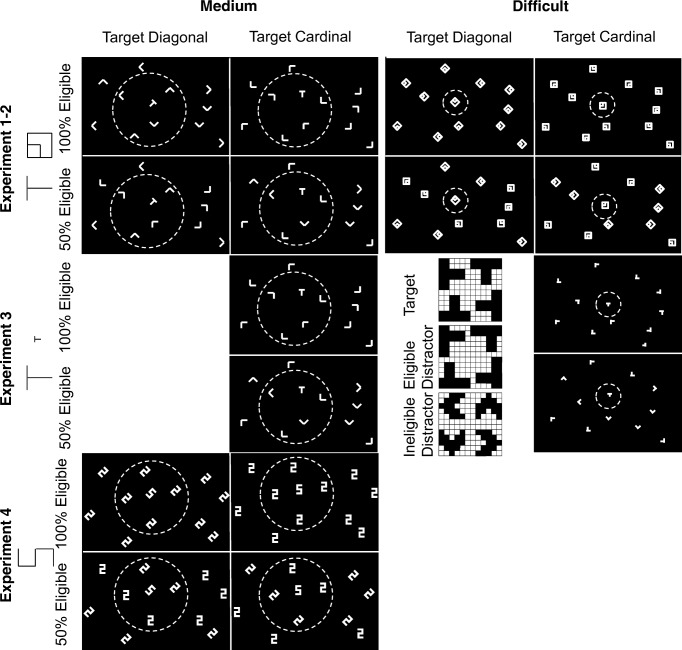
Setup for the experiments. Participants always knew the set of lines (cardinal/diagonal) used to draw the target and their exact configuration. Experiments 1 and 2: Medium search was for a T amongst Ls. Difficult search was for a configuration of squares. Experiment 3: Both medium and difficult search were for a T amongst Ls. The inset shows the detail of the difficult search items used in Experiment 3. **Inset Top:** Target (all participants were assigned one of the four versions). **Inset Middle:** Eligible distractors (all four versions appeared in each display). **Inset Bottom:** Ineligible distractors (all four versions used in each 50% eligibility display). Experiment 4: Medium search for a 5 amongst 2s. When eligibility was 100% all items in the display were created with lines from the same set as the target. When eligibility was 50%, half of the search items came from the different set. Note that with the exception of Experiment 4, the distractors always had various configurations (even in 100% eligibility). The dotted circle plotted around the target depicts the estimated size of the FVF for the task in question (Young & Hulleman, 2013)

The basic manipulation in our experiments was the following. We used two search tasks that are purely item-by-item according to the three item-based theories, but that yield different FVF sizes according to Hulleman and Olivers (2017a). For the medium search with a multi-item FVF we picked T versus L; for difficult search with a single-item FVF we picked search for a configuration of two squares (see Fig. 1). T versus L search is a prototypical example of item-by-item search according to both FIT and Guided Search. The configuration search has been used in Hulleman (2010) and Young and Hulleman (2013) and yielded slopes that were comfortably steeper than those of T versus L. We used two sets of oriented lines to construct the search items. The cardinal set contained horizontal and vertical lines, the diagonal set contained left diagonal and right diagonal lines. In the 100% eligibility condition, all items (T, Ls and squares) were constructed from a single set. In the 50% eligibility condition this changed. Now half of the items were created with lines from the cardinal set and the other half were created with lines from the diagonal set. This yielded displays where half of the items were rotated 45° relative to the other half.

Before the search trials started, participants were told which set of lines (cardinal/diagonal) was used to create the target. Because all of the distractors came from the same set in the 100% eligibility display, this information did not distinguish between target and distractors. In the 50% eligibility condition however, this information made half of the items ineligible on the basis of their orientation alone. In light of the results to come, it is probably useful to explain in detail why AET, FIT and Guided Search all predict that performance in T versus L search should improve when half of the items are rotated by 45°.

According to Guided Search 4.0 (Wolfe, 2007 p.104-106), the bottom-up activation for an individual item is based on a pairwise comparison between its response to a particular categorical channel and that of all the other items. In 100% eligibility displays (taking search for a target constructed from the lines in the cardinal set as an example) this difference will always be 0. For the ‘left’ and ‘right’ orientation channel the output is 0 for all items in the display, because none is constructed from diagonal lines. For the ‘steep’ and ‘shallow’ channel the output is maximal for all items and all pairwise comparisons will therefore again find no difference. Consequently, there is no bottom-up activation for any of the items. Moreover, none of the four orientation channels can be used for top-down guidance either. The responses for each item are identical in all four channels (either 0 or maximal). This means that picking one of the four channels will not make the target stand out more.

The situation changes for the 50% eligibility displays. Now, there will be differences in response in the orientation channels. For eligible items the response is maximal in the vertical and horizontal channel and 0 in the diagonal channels. For ineligible items this pattern is reversed: they have maximal response in the diagonal channels and 0 response in the vertical and horizontal channels. So all items (both eligible and ineligible) will have some bottom-up activation and the larger the number of nearby items that is drawn from the opposite eligibility group, the larger this bottom up activation will be. But the bottom-up activation cannot be used to reliably distinguish between eligible and ineligible items. For top-down activation the addition of ineligible items is much more consequential. The vertical (or horizontal) channel can now be used to boost the overall activation of the eligible items. The response in these channels is maximal for the eligible items and 0 for the ineligible items. By setting the weight to 1.0 for the vertical channel and to 0 for all the other channels, the overall level of activation (i.e. the sum of bottom-up activation, top-down activation and noise) will be considerably higher for eligible items than for ineligible ones. This means that eligible items will be selected earlier, and it should therefore take less time to find the T. Given that only the eligible items receive a boost in the activation map, it should also become easier to decide that there are no viable candidates left by increasing the activation threshold.^1^

**Fig. 2:**
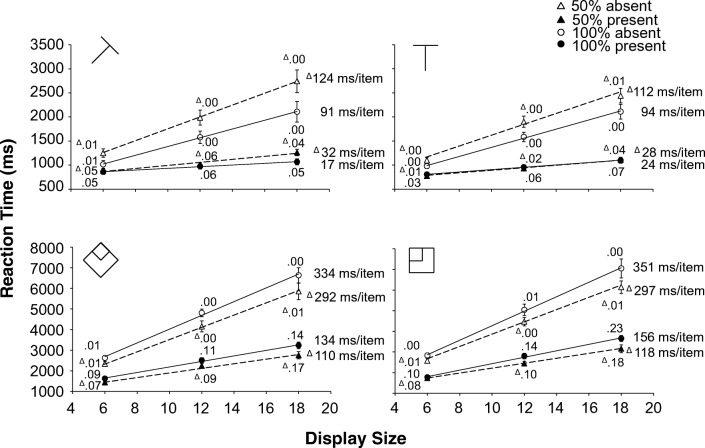
Experiment 1. Reaction times as a function of display size. Top half medium search; bottom half difficult search (please note the difference in scale. Difficult search is much slower than medium search). **Left half:** targets created with the diagonal line set. **Right half:** targets created with the cardinal line set. Dotted lines and triangles: 50% eligibility; solid lines and circles: 100% eligibility. The white symbols are absent trials, the black symbols are present trials. Next to each data point, the proportion error is given. On the side of each graph is the search slope. The error bars represent standard error of the mean. Where there appear to be no error bars, they are covered by the data point

FIT has not been specified in the same amount of computational detail as Guided Search, but the prediction of improvement is largely along the same lines. In Treisman and Sato (1990) it is acknowledged that search for a T amongst Ls in four randomly varying orientations requires item-by-item search since there is no unique feature through which inhibition of the master map can be controlled (p.476). However, when 50% of the items are rotated, the orientation map can take up this role. Now, inhibition from one (or both) of the diagonal orientation maps will reduce the activity for distractors in the master map. This effectively reduces set size and should yield better search performance.

For AET, the prediction of improvement by rotating half of the items by 45° seems counterintuitive. At first sight this manipulation appears to increase the heterogeneity of the distractors, thereby predicting a decrease in performance. However, it should be kept in mind that the crucial factor in AET is the selection weight of an item. Its size determines an item’s competition strength for entrance to VSTM. The selection weight is controlled by (1) the match of an item with the search template (with a better match resulting in a higher selection weight) and (2) weight linkage (with changes in selection weight passed on to similar items). Since Duncan and Humphreys (1992) “[…] *assume that templates specify only the relevant attributes of target stimuli”* (p.580) it seems clear that the orientation of the target will be part of the template. Consequently, only the eligible items will have a large selection weight (especially because Duncan & Humphreys, 1992, hold that special weight is given to identity on a feature and for matches with the template in particular). Therefore, the decrease in N-N similarity does not come into play, since the selection weight of non-targets is small to begin with. This also follows from Duncan and Humphreys’ (1989) suggestion that if T-N similarity is small enough, N-N similarity does not matter (p.442). Consequently, only the eligible items will have selection weights that are high enough to compete for access to VSTM, again effectively reducing set size and improving performance.

In summary, FIT, Guided Search and AET all predict improved performance in T versus L search when half of the items are made ineligible by rotating them 45°. For Guided Search and FIT this is because there is now a unique feature through which inhibition (FIT) or guidance (Guided Search) can be controlled. For AET, this is because there is now a group of distractors in the display that does not match the search template. This group will therefore not compete for entrance to VSTM and consequently will make it easier for the target to be selected. The same prediction can be derived for the difficult search task, since the theories do not make any principled distinction between different kinds of item-by-item search.

This prediction that the effect of the eligibility manipulation should be the same in medium and difficult search is even more important than the prediction of improvement itself. Specific predictions for the effect of reducing eligibility in medium and difficult search do not actually follow directly from the FVF-framework of Hulleman and Olivers, but it is in allowing for a qualitative difference between the two kinds of search that it distinguishes itself from the three item-based theories.

In total, we performed four experiments that compared the effects of reducing eligibility for the two search difficulties (see Fig. 1). The medium task was T versus L search (Experiments 1–3) or 5 versus 2 (Experiment 4), while the difficult tasks were search for a configuration of squares (Experiments 1 and 2) and search for a small T amongst small Ls (Experiment 3). Participants were shown the target in advance. So they knew which line set was used to construct the target and even the exact configuration of the target. Perhaps surprisingly, at least for FIT, AET and Guided Search, all experiments found that reducing the number of eligible items impedes performance in medium search but improves it in difficult search.

## Method

### Participants

Sixteen undergraduates of The University of Manchester participated in Experiment 1 (age range 18–21 years, 14 females, all right-handed except for one male). Eighteen undergraduates of the University of Hull were tested in Experiment 2 (age range 19–30 years, 16 females, all right-handed except for one female). Seventeen different undergraduates from the University of Hull took part in Experiment 3 (age range 18–29 years, six females, all right-handed except for two males). Thirty-two new undergraduates participated in Experiment 4 (16 from the University of Manchester: age range 18–23 years, 11 females, all right-handed except for three females and 16 from the University of Hull: age range 18–37 years, 14 right-handed females, one left-handed female and one left-handed male). All participants had normal or corrected-to-normal vision and were naïve to the purpose of the experiment. They took part in return for course credit and gave written informed consent.

Based on previous work (Hulleman, 2009, 2010; Young & Hulleman, 2013) the first three experiments were designed for N=16 (please see footnote 2 for an *a priori* power calculation).^2^ Data collection was stopped once this number was reached. Two participants were excluded from Experiment 2 due to high error rates, necessitating the recruitment of an additional two participants. One participant was excluded from Experiment 3 due to slow RTs, hence the recruitment of one extra participant. Because Experiment 4 was designed as a crucial control experiment the number of participants was doubled to 32.^3^

### Apparatus and stimuli

Software, custom-written in C, controlled stimulus presentation, response recording and eye tracking. Experiment 1 used a BenQ XL2420B LCD display controlled by a Dell Optiplex GX-620 PC with Intel 82945G Express integrated graphics. Experiments 2, 3 and 4 (Hull) used an Iiyama 454 Vision Master Pro CRT controlled by a GeForce 6800 graphics card. Experiment 4 (Manchester) used a ViewSonic VX2268WM LCD display controlled by a Quadro FX580 graphics card. The resolution was 800 x 600 pixels for all experiments.

An SR Research Ltd Eyelink 1000 tracked eye movements in Experiments 2–4. An acceleration threshold of 8,000 °/s^2^ and a velocity threshold of 30 °/s were used to detect saccades. Although participants viewed the displays binocularly, only one eye was tracked. A chin and headrest were used throughout Experiments 2, 3 and 4. At the start of each block a calibration and validation were performed. During each break, this calibration was revalidated. If necessary, a new calibration followed.

Figure 1 illustrates typical displays across the four experiments. The search items in Experiments 1 and 2 consisted of white lines (0.1° x 0.96°) on a black background. In the medium search condition, the target was a T amongst Ls (0.96° x 0.96°). In the difficult condition, the target was a particular configuration of a smaller square (0.48° x 0.48°) embedded within a larger square (0.96° x 0.96°). Items drawn with lines from the cardinal set could have four different orientations (-90°, 0°, 90° and 180°, since a horizontal and a vertical line were used to construct them) as could items drawn with lines from the diagonal set (-135°,-45°, 45° and 135°; drawn with two diagonal lines).

In Experiment 3, medium search was the same as before, but difficult search now also involved T versus L. However, the items were much smaller (.24°x.24°). Additionally, the eligible L distractors were made to resemble the target T more closely by moving the upper arm closer to the centre (see inset of Fig. 1). Only the cardinal line set was used to create the target.

In Experiment 4, participants searched for a 5 amongst 2s. The items were rectangular (.67° x 1.34°) with an aspect ratio of 1:2. All distractor 2s drawn from the same line set were identical.

In all four experiments, search items were presented in a virtual rectangle (29.0° x 19.3°). Locations within the rectangle were randomly selected, but a minimum distance of 1.45° between all items was enforced.

### Design and procedure

Experiments 1 and 2 employed a five-factor within-participant design, manipulating difficulty (medium, difficult), eligibility (100%, 50%), target line set (cardinal, diagonal), display size (Exp. 1: 6, 12, 18 items; Exp. 2: 12, 18) and target (present, absent, weighted 50:50). Experiment 3 dropped the target-line set factor, yielding a four-factor within-participant design: difficulty (medium, difficult), eligibility (100%, 50%), display size (12, 18) and target (present, absent, weighted 50:50). Experiment 4 dropped the difficulty factor, yielding the following four-factor within-participants design: eligibility (100%, 50%), target line set (cardinal, diagonal), display size (6, 12, 18) and target (present, absent, weighted 50:50).

Participants were instructed about the identity of the target and the line set used to construct it. All the distractors were either created with lines from the same line set as the target (100% eligibility) or half of the distractors would consist of lines from the other line set (50% eligibility), rendering them ineligible. For T versus L search (Experiments 1–3) this meant that even in 100% eligibility trials there were four different types of distractors. Similarly, there were three different types of distractors in the 100% eligibility trials of difficult search (Experiment 1–2). The target in Experiments 1 and 2 was either constructed with lines from the cardinal line set (i.e. the target consisted of a horizontal and a vertical line) or from the diagonal line set (i.e. the target consisted of two diagonal lines). Experiment 3 used a target constructed from the cardinal line set only. In the 5 versus 2 task of Experiment 4, there was only a single type of distractor for each line set. A distractor was either an upright 2 (cardinal line set) or a 2 tilted 45° clockwise (diagonal line set). Depending on the target line set condition, the 5 was always upright (cardinal line set) or tilted 45° clockwise (diagonal line set).

Participants always knew the exact arrangement of the lines constituting the target (i.e. its identity). For T versus L search (Experiments 1–3) this arrangement remained constant for each participant, but was varied between participants. This ensured compatibility with the difficult condition of Experiments 1 and 2, where the target for all participants was always a square with the smaller square in the top left corner when the cardinal line set was used and a square with the smaller square in the top corner when the diagonal line set was used. All participants in Experiment 4 searched for the same 5.

The factors were fully crossed in all four experiments. There were 960 trials in Experiment 1 and 800 trials in Experiment 2 (both 20 repetitions per cell). Experiment 3 had 400 trials (with 25 repetitions per cell) and Experiment 4 had 600 trials (with 25 repetitions per cell). Where manipulated, trials were blocked by task difficulty and target line set, counterbalanced in a Latin square.

Each trial began with a 1,000-ms blank screen, after which a fixation cross was presented for 500 ms. Next, the search display appeared and remained onscreen until response (see Fig. 1 for example displays). The participants’ task was to indicate the target’s presence or absence using the ‘Z’ and ‘M’ keys on a UK keyboard (Experiment 1) or the left or right triggers on a SideWinder gamepad (Experiments 2, 3 and 4).

Participants received at least ten practice trials. If the percentage correct was lower than 90% or reaction speed was considered too slow, they would receive another ten practice trials. There were self-paced breaks after every 40 trials in the medium task of Experiments 1 and 2 (50 in Experiment 3; 25 in Experiment 4) and every 20 trials in the difficult task of Experiments 1 and 2 (25 in Experiment 3).

## Results

In the following analyses, we will focus on the interactions involving eligibility and difficulty, since they track whether the effect of reducing eligibility from 100% to 50% depends on the difficulty of the search task. For the RTs and fixation counts of Experiments 1 and 2, we will only report the t-tests for the interaction contrast (Medium_100%_- Medium_50%_)−(Difficult_100%_-Difficult_50%_) for each combination of display size and target.^4^ Whenever this contrast is significant, it indicates that there is a difference in the effect of introducing ineligible items between medium and difficult search. For the slightly simpler designs of Experiments 3 and 4 we will use the step-down analysis of the Greenhouse-Geisser corrected ANOVAs proposed by Maxwell and Delaney (1990). For the error analyses, the main focus will be on whether there are any signs that the RT results may have been caused by speed-accuracy trade-off.

## Experiment 1

### Data cleaning

Trials where the RTs were outside 2.5 SDs from the cell mean^5^ (2.0%) were excluded. All remaining trials were used in the error analysis, but only correct trials were used in the RT analysis. The RTs and error rates are shown in Fig. 2.

### Reaction times

For the absent trials the interaction contrast (Medium_100%_- Medium_50%_)−(Difficult_100%_-Difficult_50%_) was significant for all three display sizes: *t*(15)=7.290, *p*<.001, *d*=1.823; *t*(15)=6.634, *p*<.001, *d*=1.658 and *t*(15)=6.479, *p*<.001, *d*=1.620 for 6, 12 and 18 items, respectively. In the medium search task, RTs for 50% eligibility were always *slower* than for 100% eligibility, whereas for the difficult search task 50% eligibility was always *faster* (all *t*(15)’s>4.190, all *ps*<.001, all *d*’s>1.047).

For the present trials the same interaction contrast was also significant for all three display sizes: *t*(15)=2.474, *p*=..026, *d*=.618; *t*(15)=4.135, *p*<.001, *d*=1.034 and *t*(15)=5.275, *p*<.001, *d*=1.319 for six, 12 and 18 items, respectively. Here again, RTs for 50% eligibility were always *faster* than for 100% eligibility in difficult search (all *t*(15)’s>2.837 all *ps*<.013, all *d*’s>.709). But in medium search, it was only in 18 items displays that 50% eligibility was *slower* than 100% eligibility, *t*(15)=3.506, *p*=.003, d=.877. For the smaller display sizes (6 and 12), there was no effect of eligibility (ps>.62).

### Error rates

Crucially, a five-way repeated-measures ANOVA (difficulty, eligibility, target line set, display size and target) on the proportion error did not find any effects involving the interaction between difficulty and eligibility (all *ps*>.270). There was a main effect of eligibility *F*(1, 15)=6.81, *p*<.020, η_p_^2^=.312 indicating fewer errors for 50% eligibility displays than for 100% eligibility displays (.040 vs. .048) and a two-way interaction between eligibility and target *F*(1, 15)=8.56, *p*<.015, η_p_^2^=.363 indicating that this error advantage for 50% eligibility displays was larger in present trials (.075 vs. .093) than in absent trials (.005 vs. .003).

## Discussion

The important result is that there was a difference in the effect of adding ineligible items. For difficult search performance improved, in line with the predictions of the three item-based theories. But performance in T versus L search actually deteriorated (with slower RTs and steeper slopes) when eligibility was reduced to 50%. This goes against the prediction of AET, GS and AET that we should see similar effects in medium and difficult search. However, before we can conclude that this is indicative of a qualitative difference in the way medium and difficult search are conducted, we have to exclude some alternative explanations.^6^ One alternative explanation, derived from Guided Search 4.0, is that the difference between medium and difficult search is due to changes in the time it takes to decide whether a particular item is a target or a distractor.

As stated in the introduction, the parallel diffusor in Guided Search 4.0 is assumed to take 150–300 ms to reach a decision boundary. It may then be the case that the improvement in performance for difficult search is due an increase in the speed to reach this boundary for ineligible items. This would selectively improve the RTs for the 50% eligibility condition in difficult search. Please note that although this proposal does explain the difference between medium and difficult search observed in Experiment 1, it does so at the considerable cost of eliminating guidance by orientation. The proposal holds that selection is essentially random for both levels of eligibility in both search tasks, but because it is easier to reject ineligible items in difficult search, an advantage for 50% eligibility emerges.

To test this explanation, we ran a replication of Experiment 1 where eye movements were recorded. If the faster RTs in 50% eligibility for difficult search are indeed due to faster rejection of ineligible items rather than avoidance of ineligible items, we would expect the number of fixations to remain constant: the same number of items is fixated as in 100% eligibility (both eligible and ineligible items are fixated, since orientation is not used for guidance), but the time it takes to reach a decision is shorter when the fixated item in question is ineligible. If ineligible items are actually avoided in difficult search (and there is therefore guidance by orientation), the number of fixations will follow the RTs and also become smaller. Avoidance of ineligible items means that fewer fixations are necessary to reach a decision.^7^ Given that the results of Experiment 1 go against the predictions of the three main theories of visual search, Experiment 2 is also useful as a replication in its own right.

## Experiment 2

### Data cleaning

**Fig. 3:**
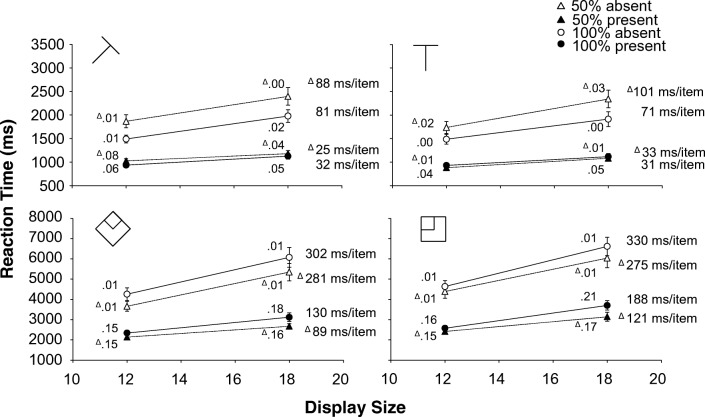
Experiment 2. Reaction times as a function of display size. Top half medium search; bottom half difficult search (please note the difference in scale; difficult search is much slower than medium search). **Left half:** targets created with the diagonal line set. **Right half:** targets created with the cardinal line set. Dotted lines and triangles: 50% eligibility; solid lines and circles: 100% eligibility. The white symbols are absent trials, the black symbols are present trials. Next to each data point, the proportion error is given. On the side of each graph is the search slope. The error bars represent standard error of the mean. Where there appear to be no error bars, they are covered by the data point

**Fig. 4:**
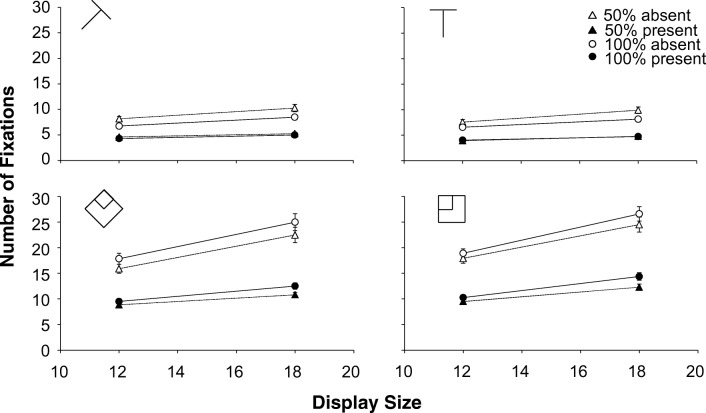
Experiment 2. Number of fixations as a function of display size. Top half medium search; bottom half difficult search. **Left half:** targets created with the diagonal line set. **Right half:** targets created with the cardinal line set. Dotted lines and triangles: 50% eligibility; solid lines and circles: 100% eligibility. The white symbols are absent trials, the black symbols are present trials. The error bars represent standard error of the mean. Where there appear to be no error bars, they are covered by the data point

Two participants were omitted from the analysis; one because they recorded 100% errors in one of the cells of the analysis, the other because their overall error rate was 2.5 SDs higher than the average of the other participants. Another 1.6% of the trials were excluded because the RTs were further than 2.5 SDs from the cell mean. All remaining trials were used in the error analysis, but only correct trials were used in the RT and eye-movement analysis. The RTs and error rates are shown in Fig. 3, the fixation counts in Fig. 4.

### Reaction times

For the absent trials the interaction contrast (Medium_100%_- Medium_50%_)−(Difficult_100%_-Difficult_50%_) was significant for both display sizes: *t*(15)=6.424, *p*<.001, *d*=1.606 for 12 items and *t*(15)=7.398, *p*<.001, *d*=1.850 for 18 items. In the medium search task, RTs for 50% eligibility were always *slower* than for 100% eligibility, whereas for the difficult search task 50% eligibility was always *faster* (all *t*(15)’s>4.640, all *ps*<.001, all *d*’s>1.160).

For the present trials the interaction contrast was significant for both display sizes as well: *t*(15)=3.250, *p*=.005, *d*=.812 for 12 items and *t*(15)=4.107, *p*<.001, *d*=1.027 for 18 items. But although 50% eligibility trials were always *faster* than 100% for difficult search (t(15)’s>2.900, ps<.011, d’s>.725), there was no effect of eligibility for medium search: t(15)’s<1.360, ps>.194, d’s<.340).

### Error rates

A five-way repeated-measures ANOVA (difficulty x eligibility x target line set x display size x target) on the error rates revealed no sign of speed-accuracy trade-off. There were no interactions involving both difficulty and eligibility. The only significant effect involving eligibility was a three-way interaction between target line set, eligibility and target *F*(1, 15)= 8.75, *p*<.01, *η*_p_^2^=.368. This reflects that the error rates for targets from the diagonal line set were essentially identical for 50% and 100% eligibility on both target-absent and target-present trials, but that for targets from the cardinal line set the error rates increased by 1 percentage point on target-absent trials when eligibility dropped to 50%, but decreased by 3 percentage points on target-present trials when eligibility was reduced to 50%.

To establish that the faster responses for the difficult 50% eligibility condition were indeed due to avoidance of ineligible items, rather than faster rejection of ineligible items once fixated, we analysed the number of fixations. If ineligible items were avoided, there should be fewer fixations for 50% eligibility. If ineligible items were only processed faster, the number of fixations should not depend on eligibility.

### Number of fixations

For the absent trials, the interaction contrast was significant for both display sizes: *t*(15)=8.000, *p*<.001, *d*=2.000 for 12 items and *t*(15)=7.922, *p*<.001, *d*=1.981 for 18 items. For medium search there were always around 1.5 *more* fixations in 50% eligibility than for 100% (t’s(15)>6.740, *ps*<.001, d’s>1.685), but for difficult search there were always around 1.9 *fewer* fixations in 50% eligibility: t’s(15)>4.397, *ps*<.001, d’s>1.021.

For the present trials, the interaction contrast was also significant for both display sizes: t(15)=2.772, *p*=.014, d=.693 for 12 items and t(15)=4.189, *p*<.001, d=1.047). For difficult search there were around 1.3 *fewer* fixations in 50% eligibility than 100% eligibility (*t*’s(15)>2.394, *ps*<.031, *d*’s>.598), but there was no effect of eligibility in medium search: *t*’s(15)<1.551, *ps*>.141, *d*’s<.388.

## Discussion

Experiment 2 not only replicated the results of Experiment 1, but it also found that the fixation counts tracked the RTs. In both RTs and eye movements there seems to be a qualitative difference in the effect of adding ineligible items between medium search and difficult search. It therefore does not appear to be the case that the improvement in performance in the 50% eligibility condition of difficult search is due to faster rejection of ineligible distractors. So, it is possible to use the difference between eligible and ineligible items to improve search performance in difficult search, but not in T versus L search. This qualitative difference between medium and difficult search is something that is much more in keeping with the fixation-based approach of Hulleman and Olivers (2017a) than with item-based theories like AET, FIT and Guided Search who do not make a principled distinction between the two kinds of search.

It seems that the ineligible items interfere with the computation of the summary statistics in easier search, because they are covered by the FVF. In difficult search the FVF is so small that it only covers a single item. Hence, there is no longer interference from neighbouring items (please see Fig. A1 in the supplementary material for an estimate of the size of the FVFs in this experiment). But before we can accept this interpretation, we have to exclude the alternative explanation that rather than demonstrating a qualitative difference between medium and difficult search per se, Experiments 1 and 2 merely found a difference between search for open and closed items, or between letters and non-letters. To generalize our results, we changed the difficult task. Medium search was as before, but difficult search now also involved T versus L. However, the items were much smaller: .24°x.24°.

## Experiment 3

### Data cleaning

**Fig. 5:**
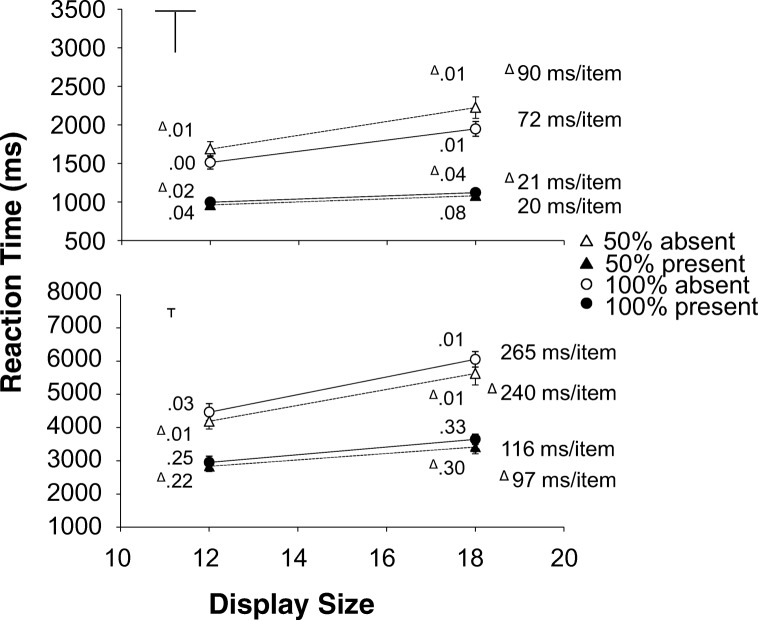
Experiment 3. Reaction times as a function of display size. Top: medium search; bottom: difficult search (please note the difference in scale. Difficult search is much slower than medium search). Dotted lines and triangles: 50% eligibility; solid lines and circles: 100% eligibility. The white symbols are absent trials, the black symbols are present trials. Next to each data point, the proportion error is given. On the side of the graphs is the search slope. The error bars represent standard error of the mean. Where there appear to be no error bars, they are covered by the data point

**Fig. 6:**
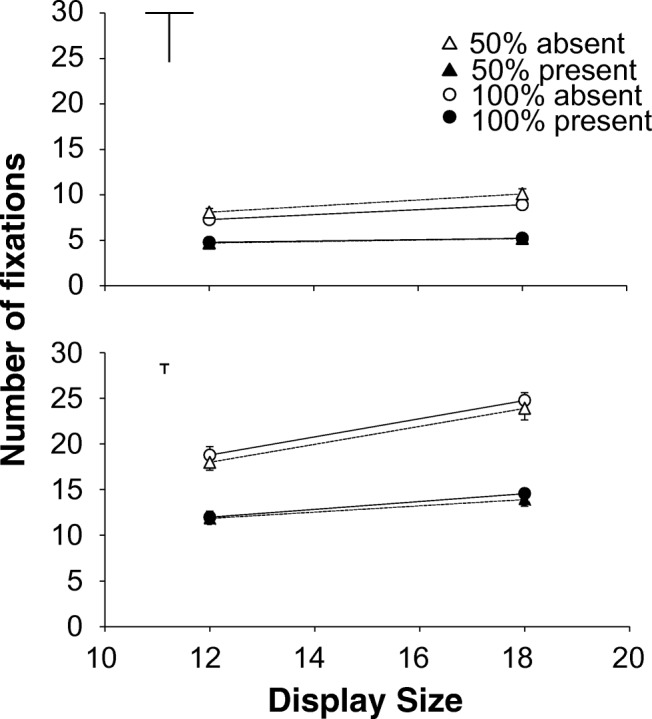
Experiment 3. Number of fixations as a function of display size. Top half medium search; bottom half difficult search. Dotted lines and triangles: 50% eligibility; solid lines and circles: 100% eligibility. The white symbols are absent trials, the black symbols are present trials. The error bars represent standard error of the mean. Where there appear to be no error bars, they are covered by the data point

One participant was removed from the analysis because their RTs were substantially slower than the group mean (2.28/2.89 SDs with their mean included/excluded). Outlier removal excluded 1.2% of the trials with RTs outside 2.5 SDs from the cell mean. All remaining trials were used in the error analysis, but only correct trials were used in the RT and eye-movement analyses. Again, the focus will be on the interactions involving difficulty and eligibility. The RTs and error rates are shown in Fig. 5, the fixation counts in Fig. 6.

### Reaction times

A four-way repeated-measures ANOVA on the RTs with difficulty, eligibility, display size and target as factors found a significant interaction between difficulty and eligibility *F*(1, 15)=54.54, *p*<.001, *η*_*p*_^*2*^=.784. with *slower* RTs for 50% eligibility in medium search, but *faster* RTs for 50% eligibility in difficult search. There was also a significant three-way interaction between difficulty, eligibility and target *F*(1, 15)=10.38, *p*<.006, *η*_*p*_^*2*^=.409. To explore further, we split the analysis by search difficulty.

For medium search, a three-way repeated-measures ANOVA (eligibility x display size x target) found a significant main effect of eligibility *F*(1, 15)=20.97, *p*<.001, *η*_*p*_^*2*^=.583 with 50% eligibility *slower* than 100% eligibility. But the two-way interaction between eligibility and target *F*(1, 15)=48.465, *p*<.001, *η*_*p*_^*2*^=.764 indicates that this held only true for absent trials (50% eligibility: 1955 ms vs. 100% eligibility: 1730 ms). For present displays there was no difference. All other interactions involving eligibility had *F*’s<3.01 and *ps*>.10.

For difficult search, a similar three-way repeated-measures ANOVA found only a main effect of eligibility *F*(1, 15)=26.86, *p*<.001, *η*_*p*_^*2*^=.642. Trials with 50% eligibility were some 263 ms *faster* than those with 100% eligibility. This main effect was not qualified by any interactions involving eligibility (all *F*’s<1.88, all *ps*>.190).

### Errors

A four-way repeated-measures ANOVA with difficulty, eligibility, display size and target on the error rates found no interactions involving both difficulty and eligibility (all *F*’s <.41 and all *ps*>.534). Probably the only noteworthy outcomes were the marginally significant main effect of eligibility *F*(1, 15)=4.29, *p*=.056, *η*_*p*_^*2*^=.222, indicating that there was a trend towards fewer errors for 50% eligibility than for 100% (.077 vs. .093), and the two-way interaction between eligibility and target *F*(1, 15)=5.98, *p*=.027, *η*_*p*_^*2*^=.285, which indicates that this trend was pronounced only for present trials (50% eligibility .146; 100% eligibility .175). For absent trials error rates were virtually identical (.009 for 50% eligibility and .010 for 100% eligibility).

### Number of fixations

A four-way repeated-measures ANOVA with difficulty, eligibility, display size and target on the number of fixations found an interaction between difficulty and eligibility *F*(1, 15)=44.36, *p*<.001, *η*_*p*_^*2*^=.747 indicating that there was a *reduction* in the number of fixations for 50% eligibility when search was difficult, but an *increase* when search was medium. Furthermore, there was a three-way interaction between difficulty, eligibility and target *F*(1, 15)=6.84, *p*=.020, *η*_*p*_^*2*^=.313. Again, we performed separate analyses for medium and difficult search

For medium search, a three-way repeated-measures ANOVA (eligibility, display size, target) yielded a main effect of eligibility *F*(1, 15)=20.642, *p*<.001, *η*_*p*_^*2*^=.579 with *more* fixations for 50% than for 100% eligibility. However, the interaction between eligibility and target *F*(1, 15)=53.241, *p*<.001, *η*_*p*_^*2*^=.780 shows that there were only *more* fixations for 50% eligibility in absent trials (50% eligibility: 9.1 fixations; 100% eligibility: 8.1 fixations). For present trials there was no effect of eligibility. The other effects involving eligibility had *ps*>.120 and *F*’s<2.70.

For difficult search, a similar three-way repeated-measures ANOVA yielded a main effect of eligibility *F*(1, 15)=10.19, *p*=.006, *η*_*p*_^*2*^=.404, with *fewer* fixations for 50% eligibility than for 100% eligibility (16.9 and 17.9 fixations, respectively). All other effects involving eligibility had *F*’s<.65 and *ps*>.430.

## Discussion

Experiment 3 replicated the results found in Experiments 1 and 2: the 50% eligibility displays yielded improved performance (in terms of search slopes and RTs) for both target-absent and target-present trials when search was difficult, but deterioration in performance for the target-absent trials of medium search. This outcome suggests that the qualitative difference between medium and difficult search found in our earlier experiments is not the consequence of differences between search for letters and search for closed shapes. Rather, it seems to be due to search difficulty per se (please see Fig. A1 in the supplementary material for an estimate of the size of the FVFs in this experiment).

Nevertheless, proponents of item-based theories may still argue that our results for medium search depend on the particular items that we have used so far. Specifically, our Ts and Ls have an aspect ratio of 1:1. This in itself might be considered to be the cause of the detrimental effect of reducing the number of eligible items in medium search: although the individual line segments of the Ts and Ls have a clear orientation, the orientations of the Ts and Ls themselves may be less clear. This could possibly impede guidance by orientation, since the orientation signal coming from the items simply is not strong enough to be effective. Hence, the increase in heterogeneity due to the introduction of ineligible items wins out and visual search performance is impeded in medium search. In addition, maybe the detrimental effect of reduced eligibility we found for T versus L will not generalize to other kinds of medium search, like 5 versus 2. To exclude these alternative explanations, we ran a final experiment.

In Experiment 4, participants searched for a 5 amongst 2s. Importantly, the items had an aspect ratio of 1:2, giving not only the constituting lines but also the items themselves a clear orientation. Moreover, 100% eligibility displays had only a single kind of distractor 2, drawn from the same line set as the target. 50% eligibility displays had two kinds of distractor 2: one drawn from the cardinal line set and one drawn from the diagonal line set. This contrasts with our previous T versus L displays where 100% eligibility displays had four kinds of distractor L and 50% eligibility displays had eight kinds of distractor L. This increase in homogeneity of the distractors should further allow performance for 50% eligibility displays to improve in 5 versus 2 search. Not only should it now be clearer that all ineligible items cannot be the target (with constituent lines drawn from a different line set and with a different overall orientation as well), they are also clearly the same, making them easier to avoid if search is really item-based.

The item-based theories therefore predict that search performance in 50% eligibility displays should be better than in 100% eligibility displays. In contrast, the prediction of the FVF-based account of Hulleman and Olivers (2017a) for this 5 versus 2 experiment remains that the introduction of ineligible items will harm search performance, because the ineligible items interfere with the computation of the summary statistics across the FVF.

## Experiment 4

### Data cleaning

**Fig. 7:**
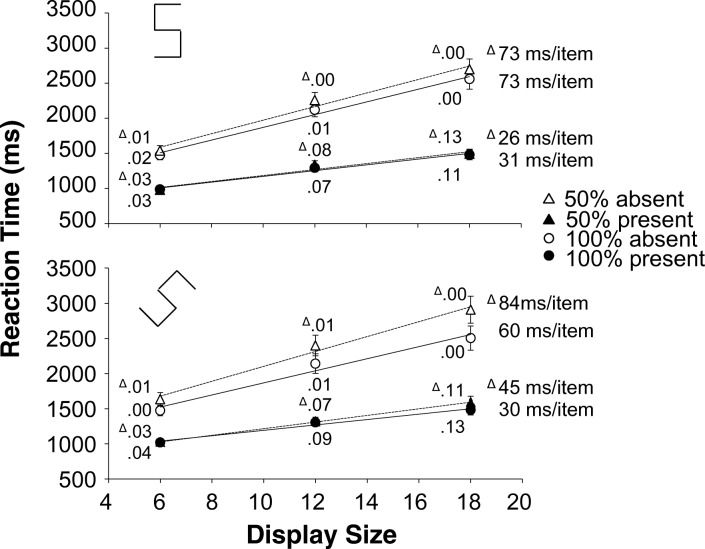
Experiment 4. Reaction times as a function of display size. Top: Target 5 created with cardinal line set; bottom: Target 5 created with diagonal line set. Dotted lines and triangles: 50% eligibility; solid lines and circles: 100% eligibility. The white symbols are absent trials, the black symbols are present trials. Next to each data point, the proportion error is given. On the side of the graphs is the search slope. The error bars represent standard error of the mean. Where there appear to be no error bars, they are covered by the data point

**Fig. 8:**
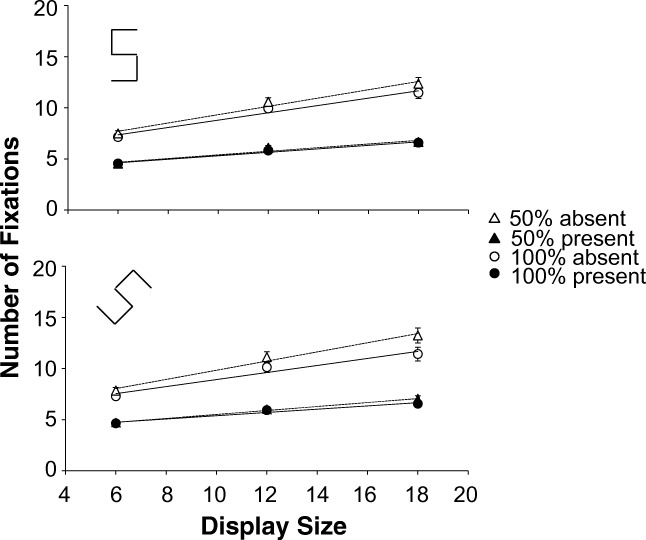
Experiment 4. Fixations as a function of display size. Top: Target 5 created with cardinal line set; bottom: Target 5 created with diagonal line set. Dotted lines and triangles: 50% eligibility; solid lines and circles: 100% eligibility. The white symbols are absent trials, the black symbols are present trials. The error bars represent standard error of the mean. Where there appear to be no error bars, they are covered by the data point

All participants were included in the analysis. Outlier removal excluded 1.2% of the trials with RTs outside 2.5 SDs from the cell mean. All remaining trials were used in the error analysis, but only correct trials were used in the RT and eye-movement analyses. Again, the focus will be on the main effect of and interactions involving eligibility. The RTs and error rates are shown in Fig. 7, the fixation counts in Fig. 8.

### Reaction times

A four-way (eligibility x target line set x display size x target) repeated-measures ANOVA on the RTs found a host of significant effects. Importantly, there were several three-way interactions that involved eligibility: an interaction between eligibility, target line set and display size *F*(1.720, 53.330)=5.192, *p*=.012, *η*_p_^2^=.142; an interaction between eligibility, target line set and target *F*(1, 31)=7.633, *p*=.010, *η*_p_^2^=.198; and an interaction between eligibility, display size and target *F*(1.958, 60.702)=3.750, *p*=.030, *η*_p_^2^=.108. Because of these interactions, the analysis was split by target presence.

For target-present trials, a three-way repeated-measures ANOVA (eligibility x target line set x display size) did not find any effects involving eligibility, although there were some trends towards *slower* RTs for 50% eligibility indicated by the main effect of eligibility *F*(1, 31)=3.937, *p*=.056, *η*_p_^2^=.113 and the interaction between eligibility and display size *F*(1.952, 60.504)=2.789, *p*=.071, *η*_p_^2^=.083. All other *p*s>.200 and all other *η*_p_^2^<.051.

For target-absent trials a similar three-way repeated-measures ANOVA found a main effect of eligibility *F*(1, 31)=94.546, *p*<.001, *η*_p_^2^=.753 with RTs for the 50% eligibility display *slower* than 100% eligibility displays (2240 ms vs. 2046 ms). This was the case for every combination of target line set and display size (all t’s(31)>3.203, all *ps*<.004, all d’s>.566), despite the presence of several qualifying interactions: a two-way interaction between eligibility and target line set *F*(1, 31)=20.693, *p*=.001, *η*_p_^2^=.400, a two-way interaction between eligibility and display size *F*(1.773, 54.957)=17.954, *p*<.002, *η*_p_^2^=.367 and a three-way interaction between eligibility, target line set and display size *F*(1.865, 57.825)=4.982, *p*=.012, *η*_p_^2^=.138.

### Errors

An eligibility x target line set x display size x target repeated-measures ANOVA on the error proportions found a solitary effect involving eligibility: the three way interaction between eligibility, target line set and target *F*(1, 31)=4.317, *p*=.046, *η*_p_^2^=.122 (all other *p*s>.165, all other *η*_p_^2^<.058). This three-way interaction reflects that for absent trials with a target from the diagonal line set participants made fewer errors on 100% eligibility trials (.005) than on 50% eligibility trials (.009): *t*(31)=2.054, *p*=.048, *d*=.363. There was no effect of eligibility for any other combination of target line set and target (all *ps*>.230, all *d*’s<.215).

### Fixations

An eligibility x target line set x display size x target repeated-measures ANOVA on the fixations found a host of significant effects. There were several three-way interactions involving eligibility: an interaction between eligibility, target line set and display size *F*(1.755, 54.404)=6.174, *p*=.004, *η*_p_^2^=.166; an interaction between eligibility, target line set and target *F*(1, 31)=4.744, *p*=.037, *η*_p_^2^=.133; and an interaction between eligibility, display size and target *F*(1.908, 59.151)=8.144, *p*=.001, *η*_p_^2^=.208. As with the RTs, the analysis was split by target presence.

For target-present trials, a three-way repeated-measures ANOVA (eligibility x target line set x display size) found a main effect of eligibility *F*(1, 31)=6.350, *p*=.017, *η*_p_^2^=.17 (100% eligibility trials had *fewer* fixations than 50% eligibility : 5.67 vs. 5.83 ). None of the interactions involving eligibility was significant (all *ps* >=.100, all *η*_*p*_^*2*^<.073).

For target-absent trials a similar three-way repeated-measures ANOVA also found a main effect of eligibility *F*(1, 31)=117.529, *p*<.001, *η*_p_^2^=.791 with *fewer* fixations for the 100% eligibility displays than 50% eligibility displays (9.56 vs.10.44). This held true for all combinations of line set and display size (all *t*’s(31)>4.307, all *ps* <.001, all *d*’s>.761), even though there were several qualifying interactions: a two-way interaction between eligibility and target line set *F*(1, 31)=13.158, *p*=.001, *η*_p_^2^=.298; a two-way interaction between eligibility and display size *F*(1.726, 53.496)=29.311, *p*<.001, *η*_p_^2^=.486 and a three-way interaction between eligibility, target line set and display size *F*(1.867, 57.879)=5.694, *p*=.007, *η*_p_^2^=.155.

## Discussion

The outcomes of Experiment 4 are in line with the results from the previous experiments, but generalize them in several ways. Despite the change to a 5 versus 2 task, we still found that introducing ineligible items impedes search performance on absent trials. Our previous results for medium search were therefore not critically dependent on the T versus L task used. Moreover, the deterioration in search performance was found even though the search items had a 1:2 aspect ratio. So, even the presence of a clear orientation signal was not enough to provide evidence of guidance by orientation. In addition, the 5 versus 2 displays used in Experiment 4 were much more homogeneous than the T versus L displays of Experiments 1–3, since all distractors drawn from the same line set were identical.

According to the item-based theories we should have found improved performance in the 50% eligibility condition, since it should have been easy to exclude the ineligible items. Not only were they constructed with lines from the wrong line set, but their overall orientation was wrong as well. However, search performance on the absent trials deteriorated when ineligible items were introduced, while search performance on present trials did not really change. It therefore seems that items are not selected individually in medium search. This kind of outcome is much more in keeping with the fixation-based framework of Hulleman and Olivers (2017a) than with AET, Guided Search or FIT. The ineligible items really do appear to interfere with the computation of summary statistics across items that fall within the FVF.

Even though the results for medium search (T vs. L and 5 vs. 2) have been consistent across our four experiments, they may strike the reader as surprising and maybe even as some kind of special case. Thus, before we turn to a discussion of the implications of our results for AET, Guided Search, and FIT, we would like to point out that we are actually not the first to encounter performance deficits when guidance by orientation is expected.

For instance, Treisman (2006) tested search for a plus consisting of a blue-vertical line and a green-horizontal line. The target was surrounded either by conjunction distractors (blue-horizontal and green-vertical plusses) or by feature distractors. The feature distractors could be *orientation* distractors where the plus was tilted 20 ° to the left or right or *colour* distractors, where one of the lines of the plus was purple. Treisman’s hypothesis was that there should be an advantage for feature surrounds, since it should be possible to use the distinctive non-target feature (either orientation or colour) to suppress the distractors, making it easier to find the target. However, contrary to expectation, there was no overall effect of surround. Closer inspection revealed that only the colour feature surround yielded an advantage. This led Treisman (2006) to remark that *“there may be something special that makes orientation feature distractors in the surround harder to suppress than colour or shape feature distractors and as hard as conjunction distractors”* (p.422). In our *General discussion* we attempt to address what this “something special” might be.

Poisson and Wilkinson (1992) ran experiments where participants searched for a conjunction target (e.g. a red-horizontal line amongst green-horizontal and red-vertical distractors). The critical manipulation involved the number of each type of distractor. Search displays varied from overwhelmingly red (with just one green-horizontal distractor and all others red-vertical) to overwhelmingly green (with just one red-vertical distractor and all others green-horizontal). They found that, on target-absent trials, participants performed better when the target was one of the two red items in an overwhelmingly green display than when the target was one of the two horizontal items in an overwhelmingly red display. According to Poisson and Wilkinson (1992) this suggested that participants prefer to use colour, rather than orientation, to select the set of items in which to search.

Poisson and Wilkinson (1992) found a similar preference when search was for a conjunction of colour and size. Most interesting though was performance in search for a conjunction of orientation and size. Here, without the possibility of guidance by colour, RTs were much slower (especially on target-absent trials) and error rates were higher. Moreover, there was no consistent preference across participants for the feature to use for segmentation of the search display. In line with our current results, this result suggests that the participants in Poisson and Wilkinson (1992) also experienced difficulties in selecting by orientation.

## General discussion

In all experiments reported here, performance in the difficult task (where tested) improved when eligibility was reduced to 50%. Both for target-present and target-absent trials, RTs became faster and slopes became shallower. For medium search however, performance clearly deteriorated on target-absent trials and did not improve on target-present trials. Experiments 2 and 3 established that this qualitative difference between medium search and difficult search was also reflected in the number of fixations and was not due to a difference between search for letters and search for closed shapes. Rather, it seems to reflect an effect of the difficulty of the search task per se. Since all three item-based approaches predict qualitatively similar results for medium and difficult search, the current results present them with an anomaly.

Both FIT and Guided Search assume that search for T versus L^8^ is already item-by-item, so there seems to be no room for the qualitative difference between medium and hard search found here. Moreover, as explained in the introduction, reducing the number of eligible items should have improved performance in T versus L search, not impeded it.

There is therefore a two-pronged problem for FIT and Guided Search. First, there is the issue of adapting the model architecture to allow for T versus L search to become slower when ineligible items are introduced. For FIT, this could be achieved by allowing cross talk between the different orientation feature maps. For Guided Search 4.0 it may be possible to adapt the weighting of the bottom-up activation to drown out any top-down guidance by orientation.

The second challenge is to ensure that these architectural adaptations do not eliminate the RT improvement that we did observe in difficult search. This seems to require some more fundamental changes to the models. One option would be the addition of a shape channel (currently not implemented in Guided Search). Such a channel may be able to capitalise on the difference between squares and diamonds in the difficult search condition of Experiments 1 and 2. However, there would be a remaining question about why the channel was unable to use the seemingly analogous difference between the Ls and chevrons of medium search, especially since this difference does appear effective in the difficult condition of Experiment 3. Moreover, reliance on shape to explain the current results may require a radical reassessment of the nature of guidance by orientation in visual search. Especially given the results of Experiment 4, which demonstrate that even a strong orientation signal is incapable of providing guidance.

Therefore, perhaps a more promising approach would be to allow the target description in T versus L search to be based on more than just a description of the T in isolation. If the relation between the target and surrounding items is also included in the target description, it might become possible to account for the influence of ineligible items in medium search. Note that this would mean that T versus L search is no longer completely unguided, even in the 100% eligibility condition. Some of the information in the extended target description will distinguish between a 100% eligibility display with a target and one without. Interestingly, such a state of affairs would actually concur with modelling results recently reported by Moran, Zehetleitner, Müller and Usher (2013). They simulated T versus L search in their Competitive Guided Search model and found that allowing for some guidance yielded a better fit with experimental data than a complete lack of guidance.

For AET to accommodate the task dependence of the effect of introducing ineligible items, it would have to be argued that for T versus L and 5 versus 2 the reduction in N-N similarity outweighed the reduction in T-N similarity, whereas in difficult search it was the other way around. But, for this account to work, it appears to be necessary that ineligible items in T versus L and 5 versus 2 search still “hit” the template, whereas this was no longer the case for the ineligible items in difficult search. One way to achieve this would be to argue that orientation was only part of the search template in difficult search. That would ensure that all of the distractors in T versus L and 5 versus 2 search compete for access to VSTM, irrespective of eligibility. However, an argument would be needed to explain why orientation was not part of the search template in medium search; especially in the case of 5 versus 2, where the aspect ratio was 1:2.

Perhaps a more elegant way would be to allow that the template specification in T versus L and 5 versus 2 search was not merely based on the target itself, but also on its relationship with surrounding distractors. This would enable the distractors to have an influence.

The fundamental problem that AET, Guided Search and FIT appear to have in common is the assumption that the target description is always based on the properties of the target in isolation. Irrespective of search difficulty, the relation of the target with its neighbours is never part of the target description. Consequently, the information that is compared against this description has to be based on information derived from a single item as well and the properties of neighbouring items will therefore play a limited role at best.^9^ The use of a pure target template in all three models means that they have difficulty capturing the influence of ineligible neighbours in medium search.

We propose that the relation between target and distractors actually is important in medium search. This suggestion receives support from another aspect of the current experiments. The detrimental effect of reduced eligibility was concentrated in the target-absent trials. Here, it seems that the mix of eligible and ineligible items gave rise to the impression that a target was present since there were orientation differences between neighbouring items. This is consistent with the idea that the relation between items was taken into account and participants were searching for deviations in this relation. The spurious orientation differences in 50% eligibility displays prevented the quicker target-absent decision that was possible in trials where all items were eligible.^10^ In the target-present trials this detrimental presence of ineligible items seems to have been balanced out by the presence of the target itself. Because the target has its own set of deviating relations with surrounding items (both eligible and ineligible) it will attract fixations in its own right (cf. Zelinsky, 1996).

A further argument for an extended target description in medium search (i.e. including relations with surrounding items) comes to the fore when we consider how inhibition is implemented in Guided Search and AET. In both, the item/template with the highest activation gets selected first. If it turns out not to be the target, it has to be inhibited to prevent it from being selected again. Both Guided Search and AET use location to apply this inhibition. In Guided Search it is only the location of the selected item that gets inhibited. In AET all the locations that are connected to the rejected template are inhibited. A clear implication of location-based inhibition of individual items is that moving them around should create a problem, since inhibited locations may no longer contain rejected distractors.

However, Hulleman (2009, 2010) demonstrated that search for T versus L is robust against motion of up to 7.2 °/s, even when the search display contained 36 items. In contrast, in difficult search (identical to the type used here in Experiments 1 and 2) search performance was worse when the items were moving. Again, this difference suggests that whereas difficult search is indeed based on the properties of single items, T versus L search is not. This becomes clear from the final experiment in Hulleman (2010). Here, participants had to decide whether there were at least five Ts among the Ls in a search display containing 12 or 18 items. The influence of item motion peaked when the number of Ts was five, was large when it was close to five (4 or 6) and was negligible when clearly not five (1, 2 or 7). This pattern indicates that it is the need to keep track of individual items that makes search vulnerable to motion. In difficult search this is always the case, but in T versus L search it only became necessary when it was important to prevent double-counting. Consequently, decisions about the presence or absence of a T among Ls in a classic search task with only a single target seem based on a process where tracking individual items is unnecessary. Rather, search is based on information obtained from multiple items simultaneously.

This conclusion also follows from Young and Hulleman (2013), who used a gaze-contingent window. When all items except the one fixated were masked, RTs, search slopes and error rates increased substantially for T versus L search. In contrast, difficult search remained mostly unaffected by the size of the gaze-contingent window. The drop in performance as a result of restricting the number of visible items to one implies that T versus L search uses information obtained from several items simultaneously when all items are visible.

The role of the relation between target and surrounding distractors in medium search means that an FVF-based approach like that of Hulleman and Olivers (2017a) allows a more natural description of the divergence between medium and difficult search. Why does orientation heterogeneity have a negative effect on performance in medium search, yet a positive effect in difficult search? As argued above, the reason seems to be that the surrounding items do have an influence in medium search, but not in difficult search. The variable size of the FVF provides an avenue to implement this difference. In medium search the FVF covers several items, allowing ineligible neighbours to wield their influence. In difficult search, the FVF covers only a single item. Hence, neighbours no longer interfere with the processing of the fixated item.

An appealing feature of an FVF-based account is that it also fits with other results that are difficult to understand from a pure target template point of view. For instance, it explains the reversal in variability of RTs going from medium to difficult search. It also explains the robustness of medium search against motion and the lack of robustness in difficult search. Interestingly, it sees all of these results as a consequence of the change in size of the FVF. Having a very small FVF minimises the influence of neighbouring items, but it makes serial fixations the driver of RTs and also makes it harder to keep track of which items have already been inspected. Having a large FVF allows the influence of neighbouring items to arise, but at the same time it makes motion in the items less of a problem and makes the RTs of target-present trials less variable than those of target-absent trials.

A further attractive feature is that an FVF-based approach is able to encompass the accounts provided by FIT, Guided Search and AET. Search with a large FVF is equivalent to what Treisman and Gelade (1980) termed feature search. Here, it is possible to determine target presence and target absence from summary statistics computed across all the items in the search display. Search with a smaller sized FVF is equivalent to what Wolfe (1994, 2007) termed Guided Search: summary statistics computed across several items allow a decision about target presence to be made. The summary statistics computed across the FVF can also be seen as implementations of the T-N and N-N similarity proposed by AET. When T-N similarity is small and N-N similarity is large, a large FVF is possible and search will be fast. When T-N similarity is large and N-N similarity is small, the FVF will be small and search will be slow and error-prone.

Finally, an FVF-based model of visual search addresses one of the other fundamental problems of AET, FIT and Guided Search: none of them has an explicit role for eye movements. They all use a representation of the visual search display that does not seem to depend on eye movements. Nor does it seem influenced by the physiological properties of the retina. For Guided Search, *“Input and initial processing of stimuli are assumed to be carried out in parallel across the entire visual field.”* (Wolfe, 1994, p.204). AET envisages *“a parallel stage of perceptual description, producing a structured representation of the input across the visual field and at several levels of spatial scale”* (Duncan & Humphreys, 1989, p.444). In FIT, *“features are registered early, automatically, and in parallel across the visual field”* (Treisman & Gelade, 1980, p.98).

## Conclusion

Our results challenge the fundamental theoretical assumption that visual search always uses a target description based on the properties of the target in isolation. While this may be the case for difficult search, our experiments suggest that for medium search (as commonly used in cognitive psychology labs) the relation between the target and distractors is important as well. Consequently, FIT, Guided Search and AET have difficulties explaining our results. An FVF-based account like Hulleman and Olivers (2017a, 2017b) seems more naturally equipped to allow for the influence of neighbouring items in medium search.

Our results also have important implications for the many studies that are predicated on the assumptions of FIT, Guided Search and AET (e.g. Becker, 2010; Eimer, 2015), for recent discussions about the nature of the target template in visual search (Olivers, Peters, Houtkamp & Roelfsema, 2011), and for our understanding of the connection between lab-based and real-world search, since the latter is frequently much more difficult than the former.

## Electronic supplementary material

ESM 1 (DOCX 106 kb)

## Footnotes

1 One of our reviewers, Garry Kong, pointed out that our argument ignores the role of singletons, both from the eligible and the ineligible line sets. Singletons can only occur in the 50% eligibility condition and could disturb the search process by attentional capture. Any singleton will probably be selected repeatedly, since it has high bottom-up activation and Guided Search has only limited memory. Consequently, due to this singleton interference, search will actually become slower in the 50% eligibility condition. We disagree for the following reasons. First, attentional capture by singletons is apparently not a problem for colour, even though Guided Search assumes that orientation and colour guide in similar ways. For instance, Fig. 2.8 in Wolfe (2014) uses a 50% eligibility display to illustrate how telling the participant that the K is red will guide attention away from the black letters to the red letters. Second, the reviewer’s argument depends on the assumption that bottom-up activation will routinely outcompete the top-down activation of the eligible items. This may be the case when the circumstances are exactly right (i.e. an item from one line set is surrounded by items from the other line set, which results in a very high bottom-up activation) but usually, when the circumstances are less than right, the bottom-up signal will lose strength, and will be drowned out by the top-down signal. Third, the cost of selecting a singleton several times will be outweighed by the gain of not having to inspect other ineligible items. By definition, an ineligible singleton will have to be surrounded by eligible items. Consequently, in the rest of the display there will be a surplus of ineligible items that either appear close together or on their own. These surplus ineligible items will score low both on bottom-up and on top-down activation and will therefore not be selected. Similar reasoning holds for an eligible singleton: it will be surrounded by ineligible singletons with low bottom-up and top-down activation, hence they will not be inspected. Finally, the 100% condition has its own specific problem that slows down search: because there is neither bottom-up nor top-down activation, it will be much more difficult to apply the activation threshold in an activation map that consists of dynamic noise. In the 50% condition there will always be a few items that have much lower activation than the rest and can therefore be excluded. So, we continue to hold that Guided Search predicts improvement in our 50% condition.

2 The crucial interaction in Experiments 1–3 is between task difficulty and eligibility. This is the interaction that tracks the difference in the effect of reducing eligibility as a function of task difficulty. As far as we can tell, there are no previous papers that investigated the relation between selection by orientation and search task difficulty. This makes it hard to come up with a power estimate for Experiment 1 based on the literature. However, because Experiment 2 is an exact replication of Experiment 1 (albeit with different display sizes and added recording of eye movements), we can use the effect sizes from Experiment 1 to estimate the power of Experiment 2 (based on N=16). The *η*_*p*_^*2*^ for the interaction between task difficulty and eligibility in Experiment 1 was .817. A priori, this means that our Experiment 2 had power >.99 to detect this interaction. This is confirmed by the *η*_*p*_^*2*^=.747 observed in Experiment 2. If we use this lower estimate of effect size to compute the power of Experiment 3, it still has power >.99 to detect this interaction between task difficulty and eligibility with N=16. The interaction between eligibility and difficulty observed in Experiment 3 had *η*_*p*_^*2*^=.784. If we look at the effect of eligibility for the two task difficulties separately, Experiment 1 found effect sizes of *η*_*p*_^*2*^=.882 and .693 for medium and difficult search, respectively. This gives Experiment 2 again power >.99 to find these effects with N=16. (The observed *η*_*p*_^*2s*^ in Experiment 2 were .741 and .648.) Using these lower estimates for Experiment 3 still yields power >.99 with N=16. The observed *η*_*p*_^*2s*^ for the effect of eligibility for medium and difficult search in Experiment 3 were .583 and .642. Experiment 4 focussed purely on eligibility in medium search. Even taking the lowest *η*_*p*_^*2*^ for medium from previous three experiments (.583) gives Experiment 4 a power of >.99 to find an effect of eligibility with N=32. The observed *η*_*p*_^*2*^ for the main effect of eligibility in the four-way ANOVA on the RTs was .739*.*

3 The important change in Experiment 4 is that all the distractors were identical (all 2s tilted clockwise or all vertical 2s) and that we strengthened the orientation signal by using a 1:2 aspect ratio. This may have led to an increased guidance by orientation, perhaps offsetting the effect of introducing ineligible items. Arguably, this could have reduced the effect size. In practice, this did not happen. We ran 16 participants in Manchester and 16 participants in Hull. Both groups showed the effects that were found in the combined data set.

4 Please note that this contrast averages over line set. But a look at the graphs of Experiments 1 and 2 suggest that any effects of line set are rather marginal. The full Greenhouse-Geisser corrected ANOVA tables are presented in the supplementary material (Tables A1–A3).

5 Here and elsewhere, cell mean refers to all observations from a unique combination of levels in the design for an individual participant.

6 We also looked for any differences between the first search block (i.e. where the participants only know the eligible items as eligible and the ineligible items as ineligible) and the second search block (i.e. where the eligible and ineligible items have switched roles). The eligibility effect is present in both blocks. It therefore does not seem to be the case that the ineligibility effect is caused by the switch from eligible to ineligible.

7 As discussed in Hulleman and Olivers (2017b), it is not the case that the visual system is blind outside of the FVF. Information outside the FVF can be used for saccadic targeting. But only within the FVF is it possible to make a reliable decision about the presence of a target.

8 In the *General discussion* we mainly focus on T versus L search, but the points made apply to 5 versus 2 search as well.

9 This focus on selection and evaluation of individual items is probably a long-term consequence of the way Treisman and Gelade (1980) originally framed the visual search problem when they proposed FIT.

10 Under our FVF-account, the presence of spurious orientation differences leads to a smaller FVF in the 50% eligibility condition and a concomitant increase in the number of fixations.
