# Supplementary material for: Medium versus difficult visual search: How a quantitative change in the functional visual field leads to a qualitative difference in performance
Source: Atten Percept Psychophys. 2019 Jul 2;82(1):118–39. doi: 10.3758/s13414-019-01787-4 (PMC6994550; doi:10.3758/s13414-019-01787-4)
Supplement: Supplementary file 1 — (DOCX 106 kb) [file 13414_2019_1787_MOESM1_ESM.docx]

**Supplementary Material**

**Table A1** Full ANOVA table for the RTs of Experiment 1

| *Factor* | *df* | *Mean Squares* | *F* | *p* | *η_p_^2^* |
| --- | --- | --- | --- | --- | --- |
| **Difficulty** | 1 | 879072229.6 | 175.411 | ***<.001*** | .921 |
|  | 15 | 5011510.429 |  |  |  |
| line set | 1 | 2027751.985 | 1.622 | *.222* | .098 |
|  | 15 | 1250251.619 |  |  |  |
| **Eligibility** | 1 | 3111265.619 | 10.413 | ***.006*** | .410 |
|  | 15 | 298774.628 |  |  |  |
| **display size** | 1.072 | 360668843.1 | 312.139 | ***<.001*** | .954 |
|  | 16.082 | 1155474.291 |  |  |  |
| **Target** | 1 | 390260640.8 | 216.631 | ***<.001*** | .935 |
|  | 15 | 1801502.95 |  |  |  |
| **difficulty * line set** | 1 | 6328282.706 | 7.363 | ***.016*** | .329 |
|  | 15 | 859461.329 |  |  |  |
| **difficulty * eligibility** | 1 | 18937832.93 | 67.090 | ***<.001*** | .817 |
|  | 15 | 282275.73 |  |  |  |
| **difficulty * display size** | 1.074 | 108219156.6 | 91.354 | ***<.001*** | .859 |
|  | 16.11 | 1184616.753 |  |  |  |
| **difficulty * target** | 1 | 86289170.7 | 57.650 | ***<.001*** | .794 |
|  | 15 | 1496773.055 |  |  |  |
| line set * eligibility | 1 | 212352.992 | 1.962 | *.182* | .116 |
|  | 15 | 108215.238 |  |  |  |
| line set * display size | 1.374 | 112839.045 | .758 | *.434* | .048 |
|  | 20.607 | 148864.661 |  |  |  |
| line set * target | 1 | 21337.986 | .095 | *.762* | .006 |
|  | 15 | 225079.732 |  |  |  |
| eligibility * display size | 1.689 | 344018.302 | 3.427 | *.055* | .186 |
|  | 25.342 | 100381.81 |  |  |  |
| eligibility * target | 1 | 16875.946 | .137 | *.716* | .009 |
|  | 15 | 123129.569 |  |  |  |
| **display size * target** | 1.087 | 76577883.02 | 87.646 | ***<.001*** | .854 |
|  | 16.308 | 873718.216 |  |  |  |
| difficulty * line set * eligibility | 1 | 220326.151 | 2.032 | *.174* | .119 |
|  | 15 | 108412.952 |  |  |  |
| difficulty * line set * display size | 1.687 | 208733.608 | 1.536 | *.235* | .093 |
|  | 25.312 | 135917.276 |  |  |  |
| difficulty * line set * target | 1 | 8574.722 | .050 | *.826* | .003 |
|  | 15 | 172010.436 |  |  |  |
| **difficulty * eligibility * display size** | 1.495 | 2495431.659 | 21.163 | ***<.001*** | .585 |
|  | 22.428 | 117914.916 |  |  |  |
| **difficulty * eligibility * target** | 1 | 4004841.34 | 31.472 | ***<.001*** | .677 |
|  | 15 | 127252.382 |  |  |  |
| **difficulty * display size * target** | 1.134 | 12039328.67 | 20.853 | ***<.001*** | .582 |
|  | 17.014 | 577334.801 |  |  |  |
| line set * eligibility * display size | 1.363 | 169519.434 | 1.351 | *.271* | .083 |
|  | 20.446 | 125490.789 |  |  |  |
| line set * eligibility * target | 1 | 22790.688 | .418 | *.528* | .027 |
|  | 15 | 54568.342 |  |  |  |
| line set * display size * target | 1.603 | 40153.109 | .526 | *.559* | .034 |
|  | 24.052 | 76341.537 |  |  |  |
| eligibility * display size * target | 1.982 | 1390.72 | .046 | *.954* | .003 |
|  | 29.736 | 30468.348 |  |  |  |
| difficulty * line set * eligibility * display size | 1.337 | 20488.674 | .182 | *.746* | .012 |
|  | 20.06 | 112757.093 |  |  |  |
| difficulty * line set * eligibility * target | 1 | 8722.973 | .186 | *.672* | .012 |
|  | 15 | 46795.237 |  |  |  |
| difficulty * line set * display size * target | 1.886 | 289.783 | .004 | *.995* | 0 |
|  | 28.293 | 69870.164 |  |  |  |
| difficulty * eligibility * display size * target | 1.802 | 194408.897 | 3.006 | *.071* | .167 |
|  | 27.037 | 64682.845 |  |  |  |
| line set * eligibility * display size * target | 1.742 | 46897.915 | .784 | *.451* | .050 |
|  | 26.13 | 59786.755 |  |  |  |
| difficulty * line set * eligibility * display size * target | 1.793 | 20546.062 | .368 | *.673* | .024 |
|  | 26.901 | 55835.925 |  |  |  |

**Table A2** Full ANOVA table for the RTs of Experiment 2

| *Factor* | *df* | *Mean Squares* | *F* | *p* | *η_p_^2^* |
| --- | --- | --- | --- | --- | --- |
| **Difficulty** | 1 | 7.87E+08 | 206.642 | ***<.001*** | .932 |
|  | 15 | 3809233 |  |  |  |
| **line set** | 1 | 5753485 | 5.330 | ***.036*** | .262 |
|  | 15 | 1079538 |  |  |  |
| **Eligibility** | 1 | 1963982 | 9.184 | ***.008*** | .380 |
|  | 15 | 213846.3 |  |  |  |
| **display size** | 1 | 85536355 | 117.934 | ***<.001*** | .887 |
|  | 15 | 725291.1 |  |  |  |
| **Target** | 1 | 3.34E+08 | 115.759 | ***<.001*** | .885 |
|  | 15 | 2884672 |  |  |  |
| **difficulty * line set** | 1 | 9757691 | 8.620 | ***.010*** | .365 |
|  | 15 | 1132004 |  |  |  |
| **difficulty * eligibility** | 1 | 12737117 | 44.197 | ***<.001*** | .747 |
|  | 15 | 288191 |  |  |  |
| **difficulty * display size** | 1 | 28304672 | 94.368 | ***<.001*** | .863 |
|  | 15 | 299939.2 |  |  |  |
| **difficulty * target** | 1 | 71696297 | 62.739 | ***<.001*** | .807 |
|  | 15 | 1142774 |  |  |  |
| line set * eligibility | 1 | 3874.465 | .068 | *.797* | .005 |
|  | 15 | 56575.01 |  |  |  |
| **line set * display size** | 1 | 261281.5 | 5.621 | *.****032*** | .273 |
|  | 15 | 46479.12 |  |  |  |
| line set * target | 1 | 298575.1 | 1.085 | *.314* | .067 |
|  | 15 | 275098 |  |  |  |
| **eligibility * display size** | 1 | 407500.1 | 6.201 | *.****025*** | .292 |
|  | 15 | 65713.61 |  |  |  |
| eligibility * target | 1 | 204243.8 | 2.544 | *.132* | .145 |
|  | 15 | 80273.31 |  |  |  |
| **display size * target** | 1 | 14000033 | 91.252 | ***<.001*** | .859 |
|  | 15 | 153421.7 |  |  |  |
| difficulty * line set * eligibility | 1 | 302006 | 2.444 | *.139* | .140 |
|  | 15 | 123582.6 |  |  |  |
| difficulty * line set * display size | 1 | 185682.6 | 3.016 | *.103* | .167 |
|  | 15 | 61575.32 |  |  |  |
| difficulty * line set * target | 1 | 294826.5 | .860 | *.368* | .054 |
|  | 15 | 342754.7 |  |  |  |
| **difficulty * eligibility * display size** | 1 | 841895 | 16.922 | ***.001*** | .530 |
|  | 15 | 49751.92 |  |  |  |
| **difficulty * eligibility * target** | 1 | 2381186 | 39.389 | ***<.001*** | .724 |
|  | 15 | 60453.23 |  |  |  |
| **difficulty * display size * target** | 1 | 3498710 | 31.692 | ***<.001*** | .679 |
|  | 15 | 110395.7 |  |  |  |
| line set * eligibility * display size | 1 | 14850.81 | .858 | *.369* | .054 |
|  | 15 | 17313.29 |  |  |  |
| **line set * eligibility * target** | 1 | 230550.9 | 5.245 | ***.037*** | .259 |
|  | 15 | 43958.62 |  |  |  |
| line set * display size * target | 1 | 97113.93 | 2.383 | *.144* | .137 |
|  | 15 | 40757.14 |  |  |  |
| eligibility * display size * target | 1 | 92527 | 2.233 | *.156* | .130 |
|  | 15 | 41431.52 |  |  |  |
| difficulty * line set * eligibility * display size | 1 | 151228.3 | 4.338 | *.055* | .224 |
|  | 15 | 34863.51 |  |  |  |
| difficulty * line set * eligibility * target | 1 | 105333.7 | 2.886 | *.110* | .161 |
|  | 15 | 36502.04 |  |  |  |
| difficulty * line set * display size * target | 1 | 72920.31 | 2.963 | *.106* | .165 |
|  | 15 | 24610.25 |  |  |  |
| difficulty * eligibility * display size * target | 1 | 1585.606 | .052 | *.823* | .003 |
|  | 15 | 30597.45 |  |  |  |
| line set * eligibility * display size * target | 1 | 553.812 | .010 | *.921* | .001 |
|  | 15 | 54873.37 |  |  |  |
| difficulty * line set * eligibility * display size * target | 1 | 8311.833 | .209 | *.654* | .014 |
|  | 15 | 39770.24 |  |  |  |

**Table A3** Full ANOVA table for the fixation counts of Experiment 2

| *Factor* | *df* | *Mean Squares* | *F* | *p* | *η_p_^2^* |
| --- | --- | --- | --- | --- | --- |
| **Difficulty** | 1 | 11988.94 | 291.856 | ***<.001*** | .951 |
|  | 15 | 41.078 |  |  |  |
| line set | 1 | 34.816 | 3.164 | *.096* | .174 |
|  | 15 | 11.004 |  |  |  |
| **Eligibility** | 1 | 19.548 | 5.021 | ***.041*** | .251 |
|  | 15 | 3.893 |  |  |  |
| **display size** | 1 | 1274.663 | 159.958 | ***<.001*** | .914 |
|  | 15 | 7.969 |  |  |  |
| **Target** | 1 | 6071.584 | 163.101 | ***<.001*** | .916 |
|  | 15 | 37.226 |  |  |  |
| **difficulty * line set** | 1 | 106.62 | 8.925 | ***.009*** | .373 |
|  | 15 | 11.946 |  |  |  |
| **difficulty * eligibility** | 1 | 185.048 | 51.495 | ***<.001*** | .774 |
|  | 15 | 3.594 |  |  |  |
| **difficulty * display size** | 1 | 426.135 | 137.816 | ***<.001*** | .902 |
|  | 15 | 3.092 |  |  |  |
| **difficulty * target** | 1 | 1340.022 | 90.331 | ***<.001*** | .858 |
|  | 15 | 14.835 |  |  |  |
| line set * eligibility | 1 | .027 | .044 | *.837* | .003 |
|  | 15 | .625 |  |  |  |
| line set * display size | 1 | 3.725 | 4.139 | *.060* | .216 |
|  | 15 | .9 |  |  |  |
| line set * target | 1 | 2.358 | .713 | *.412* | .045 |
|  | 15 | 3.309 |  |  |  |
| eligibility * display size | 1 | 3.874 | 3.666 | *.075* | .196 |
|  | 15 | 1.057 |  |  |  |
| **eligibility * target** | 1 | 5.045 | 5.036 | ***.040*** | .251 |
|  | 15 | 1.002 |  |  |  |
| **display size * target** | 1 | 220.662 | 120.101 | ***<.001*** | .889 |
|  | 15 | 1.837 |  |  |  |
| difficulty * line set * eligibility | 1 | 1.959 | 1.326 | *.268* | .081 |
|  | 15 | 1.478 |  |  |  |
| difficulty * line set * display size | 1 | 2.324 | 1.960 | *.182* | .116 |
|  | 15 | 1.186 |  |  |  |
| difficulty * line set * target | 1 | 1.67 | .366 | *.554* | .024 |
|  | 15 | 4.566 |  |  |  |
| **difficulty * eligibility * display size** | 1 | 13.726 | 17.098 | ***.001*** | .533 |
|  | 15 | .803 |  |  |  |
| **difficulty * eligibility * target** | 1 | 30.257 | 38.426 | ***<.001*** | .719 |
|  | 15 | .787 |  |  |  |
| **difficulty * display size * target** | 1 | 63.097 | 34.031 | ***<.001*** | .694 |
|  | 15 | 1.854 |  |  |  |
| line set * eligibility * display size | 1 | .038 | .142 | *.712* | .009 |
|  | 15 | .269 |  |  |  |
| line set * eligibility * target | 1 | 2.077 | 2.903 | *.109* | .162 |
|  | 15 | .715 |  |  |  |
| line set * display size * target | 1 | 1.118 | 1.336 | *.266* | .082 |
|  | 15 | .837 |  |  |  |
| eligibility * display size * target | 1 | 1.511 | 1.676 | *.215* | .101 |
|  | 15 | .901 |  |  |  |
| difficulty * line set * eligibility * display size | 1 | .942 | 1.779 | *.202* | .106 |
|  | 15 | .529 |  |  |  |
| difficulty * line set * eligibility * target | 1 | 1.515 | 1.836 | *.196* | .109 |
|  | 15 | .825 |  |  |  |
| difficulty * line set * display size * target | 1 | .74 | 1.250 | *.281* | .077 |
|  | 15 | .592 |  |  |  |
| difficulty * eligibility * display size * target | 1 | .052 | .118 | *.736* | .008 |
|  | 15 | .441 |  |  |  |
| line set * eligibility * display size * target | 1 | 0 | .001 | *.982* | 0 |
|  | 15 | .882 |  |  |  |
| difficulty * line set * eligibility * display size * target | 1 | .112 | .152 | *.702* | .010 |
|  | 15 | .737 |  |  |  |

**Fig. A1** Estimates of the FVF in the 100% eligibility condition for medium and difficult search in (A) Experiment 2 and (B) Experiment 3. Black symbols: Difficult search; White symbols: medium search. Diamonds: target from the diagonal line set; Squares: target from the cardinal line set. Small symbols: display size 12; Large symbols: display size 18. The grey bars indicate the estimated radius of the FVF in the 100% eligibility condition following the method of Young and Hulleman (2013).
